# Sickness Absence Due to Specific Mental Diagnoses and All-Cause and Cause-Specific Mortality: A Cohort Study of 4.9 Million Inhabitants of Sweden

**DOI:** 10.1371/journal.pone.0045788

**Published:** 2012-09-25

**Authors:** Ellenor Mittendorfer-Rutz, Linnea Kjeldgård, Bo Runeson, Aleksander Perski, Maria Melchior, Jenny Head, Kristina Alexanderson

**Affiliations:** 1 Department of Clinical Neuroscience, Division of Insurance Medicine, Karolinska Institutet, Stockholm, Sweden; 2 Department of Clinical Neuroscience, Centre for Psychiatric Research, Karolinska Institutet, Stockholm, Sweden; 3 Stress Research Institute, Stockholm University, Stockholm, Sweden; 4 INSERM, Villejuif, France; 5 Department of Epidemiology and Public Health, University College London, London, United Kingdom; Catholic University of Sacred Heart of Rome, Italy

## Abstract

**Background:**

Despite the magnitude and increase of sickness absence due to mental diagnoses, little is known regarding long-term health outcomes. The aim of this nationwide population-based, prospective cohort study was to investigate the association between sickness absence due to specific mental diagnoses and the risk of all-cause and cause-specific mortality**.**

**Methods:**

A cohort of all 4 857 943 individuals living in Sweden on 31.12.2004 (aged 16–64 years, not sickness absent, or on retirement or disability pension), was followed from 01.01.2005 through 31.12.2008 for all-cause and cause-specific mortality (suicide, cancer, circulatory disease) through linkage of individual register data. Individuals with at least one new sick-leave spell with a mental diagnosis in 2005 were compared to individuals with no sickness absence. Hazard ratios (HR) and 95% confidence intervals (CI) were estimated by Cox regression, adjusting for age, sex, education, country of birth, family situation, area of residence, and pre-existing morbidity (diagnosis-specific hospital inpatient (2000–2005) and outpatient (2001–2005) care).

**Results:**

In the multivariate analyses, mental sickness absence in 2005 was associated with an increased risk for all-cause mortality: HR: 1.65, 95% CI: 1.47–1.86 in women and in men: 1.73, 1.57–1.91; for suicide, cancer (both smoking and non-smoking related) as well as mortality due to circulatory disease only in men. Estimates for cause-specific mortality ranged from 1.48 to 3.37. Associations with all-cause mortality were found for all mental sickness absence diagnostic groups studied.

**Conclusions:**

Knowledge about the prognosis of patients sickness absent with specific mental diagnoses is of crucial clinical importance in health care. Sickness absence due to specific mental diagnoses may here be used as a risk indictor for subsequent mortality.

## Introduction

Worldwide, mental disorders are increasing [1], [2]. By 2030, unipolar depressive disorder, for example, has been predicted to be the leading cause of DALYs (disability adjusted life years) in high-income countries [1]. Mental diagnoses rank as the leading causes of long-term sickness absence in several countries [3], [4] and sickness absence due to mental diagnoses is now a common prescription in health care [5]. Despite the magnitude of the public health problem of sickness absence due to mental diagnoses, little is known regarding long-term health outcomes.

Recent studies suggest an increased risk of overall and cause-specific mortality for individuals who were on mental sickness absence [6], [7], [8], [9], [10]. A limitation of these studies is that they were based on selected occupational cohorts, who tend to have lower mortality rates than the general population, [11] or on limited geographical areas. Therefore, it is not clear whether findings from these studies apply to all settings, and there is a need to conduct similar analyses in larger populations. In the present investigation, we used data from the entire working-age population of Sweden, thereby avoiding selection biases related to participation in health studies or into specific occupations [12].

All people above the age of 16, living in Sweden, with an income from work or unemployment benefits, who due to disease or injury have a reduced work capacity, are covered by the national sickness insurance and can receive sickness benefits [13]. After a first qualifying day, the employer pays sick pay for the first 14 days of a sick-leave spell, thereafter sickness benefit is paid by the Social Insurance Agency. Self employed have more qualifying days, and they as well as unemployed get all sickness benefits from the Social Insurance Agency. A physician certificate is required after seven days of self-certification. In this study, data on sick leave with benefits from the Social Insurance Agency was used.

Various mental diagnoses have been shown to be associated with an increased risk of all-cause and cause-specific mortality [14], [15], [16], [17], [18]. Mortality rates seem to be elevated across all treatment settings, with highest estimates for patients treated in inpatient care and lower rates for patients treated in general practice [17]. Mental sick-leave diagnoses cover a wide spectrum of mental disorders, particularly widespread mental disorders like depression and stress-related mental disorders [19]. These disorders are managed to a considerable extent in primary health care [19]. It is not known to date to which extent specific mental sick-leave diagnoses predict all-cause and cause-specific mortality independent from previous health care contacts, particularly psychiatric inpatient and outpatient health care.

The aim of this study was to examine the association of sickness absence due to specific mental diagnoses and the risk of all-cause and cause-specific mortality, when adjusting for a range of different socio-demographic factors and previous healthcare due to somatic and mental diagnoses.

**Table 1:** Descriptive statistics of 4 857 943 women and men, aged 16–64 years and living in Sweden, neither on old-age nor disability pension and without an ongoing sick-leave spell at the turn of 2004/2005.

Characteristics	Women		Men	
	N	%	N	%
Study population, all	2307056	47.5	2550887	52.5
***Sickness absence, 2005***				
No sickness absence	2056182	89.1	2384466	93.5
Sickness absence; mental diagnoses	52083	2.3	24967	1.0
Sickness absence; non-mental diagnoses	198791	8.6	141454	5.5
***Age group, years***				
16–24	448147	19.4	470087	18.4
25–34	515871	22.4	552616	21.7
35–44	526449	22.8	589279	23.1
45–54	447692	19.4	508505	19.
55–64	368897	16.0	430400	16.9
***Educational level (years)***				
Low (≤9)	426735	18.5	576202	22.6
Medium (10–12)	1039765	45.1	1221633	47.9
High (≥12)	840556	36.4	753052	29.5
***Family situation***				
Married/living with partner without children	327985	14.2	314795	12.3
Married/living with partner with children	838612	36.3	875617	34.3
Single/divorced/separated/widowed without children	696117	30.2	1048849	41.1
Single/divorced/separated/widowed with children	224648	9.7	65765	2.6
Adolescents living with parents, 16–20 years	219694	9.5	245861	9.6
***Area of residence*** 1				
Big cities	883984	38.3	940349	36.9
Medium-sized cities	814508	35.3	904671	35.5
Small cities/villages	608564	26.4	705867	27.7
***Country of birth***				
Sweden	1985456	86.1	2224802	87.2
Other Northern European countries	71399	3.1	63857	2.5
EU25 without Northern European countries	46911	2.0	48518	1.9
Rest of the world	203290	8.8	213710	8.0.4
***Cause of death, 2005–2008***				
Suicide	512	6.1	1651	9.4
Cancer	4970	58.9	5748	32.8
Smoking-related cancer	1706	20.2	2456	14.0
Non-smoking related cancer	3264	38.7	3292	18.8
Circulatory disease	1256	14.9	4729	27.0
Other causes of death	1698	20.1	5383	30.7

1 Area of residence: big cities: Stockholm, Gothenburg and Malmö; medium-sized cities: cities with more than 90 000 inhabitants within 30 km distance from the centre of the city; small cities/villages [20].

## Methods

### Ethical Statement

The study population was based on linkage of several public national registers. Ethical vetting is always required when using register data in Sweden. The ethical vetting is performed by regional ethical review boards and the risk appraisal associated with the Law on Public Disclosure and Secrecy is done by data owners. The ethical review boards can however waive the requirement to consult the data subjects (or in case of minors/children the next of kin, careers or guardians) directly to obtain their informed consent, and will often do so if the research is supported by the ethical review board and the data has already been collected in some other context. According to these standards in Sweden this project has been evaluated and approved by the Regional Ethical Review Board of Karolinska Institutet, Stockholm, Sweden.

From a cohort including all 5 750 718 individuals aged 16–64 years and living in Sweden, 31.12.2004 we excluded 443 individuals who died before 2005 or were erroneously registered as alive in 2005. We further excluded 274 516, 36 322 and 501 678 individuals with on-going sickness absence, old-age and disability pension during 2004/2005, respectively, and 79 816 individuals due to missing values on covariates (primarily due to missing information on educational level (N = 79 359)). Individuals on old-age or disability pension were excluded due to the fact that they were not at risk of the exposure, that is, sickness absence. After these exclusions, a cohort of 4 857 943 individuals (47.5% of all individuals were women) was followed prospectively from 01.01.2005 up until 31.12.2008 with respect to all-cause and cause-specific mortality.

### Register Linkage

Register data was obtained and merged for each individual from study entry (01.01.2005) up to the end of follow-up (31.12.2008) from the following three authorities:

1. Statistics Sweden: age, sex, country of birth, family situation, area of residence, educational level.
2. The Social Insurance Agency: sickness absence, disability pension.
3. The National Board of Health and Welfare: data on morbidity (hospital inpatient and outpatient care) and on mortality.

### Sickness Absence

Exposure was measured as having at least one new sick-leave spell due to mental diagnoses or non-mental diagnoses (including missing diagnoses, 19%) during 2005, as registered by the Social Insurance Agency. The main diagnosis on the sickness certificate was used. Individuals with no recorded new sick-leave spells during 2005 were used as the reference group.

**Table 2:** Crude and adjusted Hazard Ratios (HR) with 95% confidence intervals (CI) for all-cause mortality related to mental and non-mental sick-leave diagnoses, stratified by sex.

Sickness absence in 2005	All, N	Deaths	Model 0		Model 1	Model 2	Model 3	Model 4
		N, %		HR (95% CI)	HR (95% CI)	HR (95% CI)	HR (95% CI)	HR (95% CI)
***Women***								
No sickness absence	2056182	5940 (0.29)		1	1	1	1	1
Sickness absence; non-mental diagnoses^1^	198791	2194 (1.1)		4.58 (4.4–4.8)	3.96 (3.8–4.2)	3.86 (3.7–4.1)	2.55 (2.4–2.7)	2.59 (2.5–2.7)
Sickness absence; mental diagnoses	52083	302 (0.58)		2.44 (2.2–2.7)	2.4 (2.1–2.7)	2.38 (2.1–2.7)	2.04 (1.8–2.3)	1.65 (1.5–1.9)
Developmental and organic disorders^2^	115	5 (4.35)		18.55 (7.7–44.6)	11.56 (4.8–27.8)	10.78 (4.5–25.9)	6.92 (2.9–16.6)	6.07 (2.5–14.6)
Substance - related disorders (F10–F19)	534	20 (3.75)		15.81 (10.2–24.5)	13.69 (8.8–21.3)	11.24 (7.2–17.4)	7.37 (4.7–11.4)	2.99 (1.9–4.7)
Schizophrenia and non - affective psychoses^3^	323	4 (1.24)		5.17 (1.9–13.8)	5.73 (2.2–15.3)	4.95 (1.9–13.2)	3.91 (1.5–10.4)	1.41 (0.5–3.8)
Affective disorders (F30–F39)	17390	106 (0.61)		2.56 (2.2–3.1)	2.6 (2.2–3.2)	2.51 (2.1–3.0)	2.08 (1.7–2.5)	1.54 (1.3–1.9)
Neurotic and somatoform disorders 4	5411	31 (0.57)		2.41 (1.7–3.4)	3.21 (2.3–4.6)	3.01 (2.1–4.3)	2.47 (1.7–3.5)	1.83 (1.3–2.6)
Stress -related mental disorders (F43)	26464	123 (0.46)		1.95 (1.6–2.3)	1.79 (1.5–2.1)	1.83 (1.5–2.2)	1.64 (1.4–1.9)	1.54 (1.3–1.8)
Behavioural disorders (F50–F59, F90–99)	1738	10 (0.58)		2.41 (1.3–4.5)	2.55 (1.4–4.8)	2.51 (1.4–4.7)	2.28 (1.2–4.2)	1.88 (1.0–3.5)
Personality disorders (F60–F69)	108	3 (2.78)		11.7 (3.8–36.3)	20.98 (6.7–65.1)	15.6 (5.0–48.4)	9.31 (3.0–3.9)	4.4 (1.4–13.7)
***Men***								
No sickness absence	2384466	13916 (0.58)		1	1	1	1	1
Sickness absence; non - mental diagnosis^1^	141454	3144 (2.22)		4.51 (4.3–4.7)	3.51 (3.4–3.7)	3.28 (3.2–3.4)	2.11 (2.0–2.2)	2.16 (2.1–2.3)
Sickness absence; mental diagnoses	24967	451 (1.81)		3.69 (3.4–4.1)	3.58 (3.3–3.9)	3.35 (3.1–3.7)	2.72 (2.5–2.9)	1.73 (1.6–1.9)
Developmental and organic disorders^2^	130	8 (6.15)		12.83 (6.4–25.7)	9.11 (4.5–18.2)	8.11 (4.1–16.2)	5.69 (2.8–11.4)	3.63 (1.8–7.3)
Substance - related disorders (abuse/(F10–F19)	1483	102 (6.88)		14.27 (11.7–17.3)	12.17 (10.0–14.8)	8.63 (7.1–10.5)	5.44 (4.5–6.6)	1.99 (1.6–2.4)
Schizophrenia and non - affective psychoses 3	370	16 (4.32)		8.94 (5.5–14.6)	11.97 (7.3–19.5)	9.15 (5.6–14.94)	6.44 (3.9–10.5)	2.39 (1.5–3.9)
Affective disorders (F30–F39)	9374	172 (1.83)		3.74 (3.2–4.4)	3.6 (3.1–4.2)	3.35 (2.9–3.9)	2.69 (2.3–3.1)	1.71 (1.5–1.9)
Neurotic and somatoform disorders 4	2903	38 (1.31)		2.67 (1.9–3.7)	3.1 (2.3–4.3)	2.77 (2.0–3.8)	2.21 (1.6–3.0)	1.41 (1.0–1.9)
Stress- related mental disorders (F43)	9948	106 (1.07)		2.17 (1.8–2.6)	2.05 (1.7–2.5)	2.1 (1.7–2.5)	1.87 (1.5–2.3)	1.63 (1.4–1.9)
Behavioral disorders (F50–F59, F90–99)	696	9 (1.29)		2.63 (1.4–5.1)	2.52 (1.3–4.9)	2.35 (1.2–4.5)	1.96 (1.0–3.8)	1.51 (0.8–2.9)
Personality disorders (F60–F69)	63	0 (0)		−	−	−	−	−

Model 0: Crude; Model 1: Adjusted for age; Model 2: As model 1 and adj. for educational level, family situation, region, and country of birth; Model 3: As model 2 and adjusted for outpatient and inpatient care due to non-mental diagnoses; Model 4: As model 3 and adjusted for outpatient and inpatient care due to mental diagnoses;

1 Including sick-leave spells for which diagnoses are missing;

2 including F00–09, F70–89;

3 including F20–F29;

4 including F40–F42, F44–F49.

### Exposure Measures

Persons contributed with person time in three different exposure categories, starting from:

1. 01.01.2005: no sickness absence (not exposed), all individuals were in this category at study entry.
2. date of the first sick-leave spell due to a non-mental diagnosis in 2005.
3. date of the first sick-leave spell due to a mental diagnosis in 2005.

Hierarchy was applied, giving priority to sick-leave spells due to mental diagnoses, meaning that exposure to a sick-leave spell due to non-mental diagnoses was only considered if it preceded but not if it followed a spell due to mental diagnoses.

The following groups of mental sick-leave diagnoses were categorised according to the *International Classification of Diseases*, tenth revision, ICD10 (in brackets the respective codes according to ICD10 are mentioned): 1. developmental and organic disorders (F00 to F09, F70 to F89); 2. substance-related disorders (F10 to F19); 3. schizophrenia and non-affective psychoses (F20 to F29); 4. affective disorders (F30 to F39); 5. neurotic and somatoform disorders (F40 to F42, F44 to F49); 6. stress-related disorders (F43); 7. behavioural disorders (F50 to F59, F90 to F99); and 8. personality disorders (F60 to F69). Due to limited power, categories 1, 3, 7, and 8 were combined in the analyses of cause-specific mortality.

### Mortality

Causes of death were coded according to ICD10: suicide (X60–X84); cancer (C00–C97, D00–D48), smoking-related cancer (oral cavity: C00–C06, C09–C14; esophagus: C15; pancreas: C25; respiratory and intrathoracic organs: C30–C34, C38 and urinary tract: C64–C68) [7] and non-smoking related cancer; as well as circulatory disease (I00–I99).

### Covariates

Covariates, namely age, educational level, family situation, area of residence, [20] and country of birth, were measured at 31.12.2004 and were categorised as indicated in Table 1. Previous health care was categorised based on the median length (total number of days) in inpatient care 2000–2005 (no inpatient care; ≤ median length; > median length) and total number of outpatient visits, 2001–2005 (no visits; ≤ median visits; > median visits). These median values were calculated diagnosis-specific. The median for inpatient care due to mental diagnoses was 6 days, due to cancer diagnoses 5 days, and due to circulatory disease and all somatic diagnoses combined 4 days. The medians for outpatient hospital visits were as follows: cancer, cardiovascular, and mental diagnoses (1 visit) and somatic diagnoses combined (5 visits). In the analyses of all-cause mortality and suicide, adjustments regarding in- and out-patient care were related to somatic and mental diagnoses, and suicide attempt, respectively. The analyses of specific causes of death were controlled for in- and outpatient care due to mental diagnoses and the diagnoses related to the specific causes of death of interest (cancer and circulatory disease).

### Statistics

Cox proportional hazards regression models were used to calculate hazard ratios (HR) and 95% confidence intervals (CI) for all-cause and cause-specific mortality after assuring that the proportional hazard assumption was met. Censoring was done due to emigration and in the analyses of cause-specific mortality; for other causes of death than the ones of interest. Analyses were stratified by gender, in case the partial likelihood ratio test indicated a significant interaction with gender. The partial likelihood ratio test was also applied to test if sickness absence due to specific mental diagnoses improved the prediction of mortality compared to sickness absence due to all mental diagnoses combined. In order to test the possibility of misinterpretation of early signs of cancer as mental symptoms, risk estimates for cancer, were estimated with a two-year wash-out period. SPSS version 20 was used for the analyses.

## Results

Among women, 2.3% had at least one new sick-leave spell due to mental diagnoses in 2005 (Table 1). The corresponding proportion among men was 1.0%. The majority of women and men in the study population were below 45 years of age, had achieved a medium educational level, were living in big cities, and were born in Sweden. In total, 25 947 deaths were identified during the four years of follow-up; 8 436 among women and 17 511 among men. The predominant causes of death among both women and men were cancer, followed by circulatory disease and suicide (Table 1).

**Table 3:** Crude and adjusted Hazard Ratios (HR) with 95% confidence intervals (CI) for suicide related to mental and non- mental sick-leave diagnoses.

Sickness absence in 2005	Suicide		Model 0	Model 1	Model 2	Model 3	Model 4
	All, N	N, %	HR (95% CI)	HR (95% CI)	HR (95% CI)	HR (95% CI)	HR (95% CI)
No sickness absence	4440648	1766 (0.04)	1	1	1	1	1
Sickness absence; non-mental diagnoses^1^	340245	203 (0.06)	1.73 (1.49–2.00)	1.97 (1.70–2.28)	1.86 (1.60–2.15)	1.37 (1.18–1.59)	1.49 (1.28–1.73)
Sickness absence; mental diagnoses	77050	194 (0.25)	7.35 (6.33–8.53)	9.47 (8.14–11.01)	8.8 (7.57–10.24)	7.45 (6.40–8.68)	3.37 (2.86–3.97)
Substance-related disorders (F10–F19)	2017	17 (0.84)	24.99 (15.5–40.3)	20.47 (12.69–33.02)	13.83 (8.56–22.34)	9.73 (6.01–15.75)	1.8 (1.10–2.93)
Affective disorders (F30 to F39)	26764	84 (0.31)	9.17 (7.36–11.41)	11.38 (9.13–14.18)	10.39 (8.33–12.95)	8.72 (6.99–10.89)	3.64 (2.89–4.58)
Neurotic and somatoform disorders^2^	8314	24 (0.29)	8.43 (5.64–12.62)	10.66 (7.12–15.96)	9.43 (6.30–14.12)	7.81 (5.21–11.71)	3.46 (2.30–5.21)
Stress - related mental disorders (F43)	36412	51 (0.14)	4.08 (3.09–5.4)	5.59 (4.22–7.4)	5.62 (4.24–7.44)	5.00 (3.78–6.63)	3.75 (2.83–4.97)
Other psychiatric disorders	3543	18 (0.51)	14.83 (9.32–23.6)	18.42 (11.57–29.32)	15.82 (9.94–25.2)	13.00 (8.16–20.71)	3.61 (2.25–5.80)

Model 0: Crude; Model 1: Adjusted for age and sex; Model 2: As model 1 and adjusted for educational level, family situation, region, country of birth; Model 3: As model 2 and adjusted for outpatient and inpatient care due to non - mental diagnoses; Model 4: As model 3 and adjusted for outpatient and inpatient care due to mental diagnoses and suicide attempt.

1 Including sick - leave spells for which diagnoses are missing;

2 including F40–F42, F44–F49.

### All-cause Mortality

In age-adjusted analyses, women and men with a new sick-leave spell due to mental diagnoses had a 2.4- and 3.7-fold increased risk of all-cause mortality, respectively (Table 2). After additional adjustment for the remaining socio-demographic variables (education, region, country of birth, and family situation) as well as inpatient and outpatient health care, women and men had a 70% increase in mortality risk. The partial likelihood ratio test revealed that information on sick leave due to specific mental diagnoses improved the prediction of all-cause mortality compared to sick leave due to all mental diagnoses combined (p<0.001).

Hazard ratios of mortality in the age-adjusted analyses ranged from 1.8 and 2.1 for sickness absence due to stress-related diagnoses to 13.7 and 12.2 for substance-related diagnoses among women and men, respectively. Strong effects of adjustment for covariates were found for sickness absence due to substance-related diagnoses as well as schizophrenia and non-affective psychoses. Lowest effects of adjustments were seen on estimates of sickness absence due to stress-related diagnoses. In the multivariate analyses, sick-leave spells due to any of the mental diagnostic groups were associated with a significantly increased risk of all-cause mortality among women and men, with the exception of schizophrenia and non-affective psychoses among women and behavioural disorders among men.

We found significant gender differences in the age-adjusted analyses with regard to the association of sickness absence due to mental diagnoses (p<0.001), and specifically due to affective diagnoses (p = 0.001), with all-cause mortality. These significant interaction effects were lost when adjusting for health care.

**Table 4:** Crude and adjusted Hazard Ratios (HR) with 95% confidence intervals (CI) for cancer mortality, smoking and non-smoking related, in relation to mental and non-mental sick-leave diagnoses.

Sickness absence in 2005	Death	Model 0	Model 1	Model 2	Model 3
	N, %	HR (95% CI)	HR (95% CI)	HR (95% CI)	HR (95% CI)
***All cancer deaths***					
No sickness absence	7070 (0.16)	1	1	1	1
Non-mental diagnoses^1^	3475 (1.02)	7.83 (7.52–8.16)	6.05 (5.8–6.3)	3.16 (3.03–3.31)	1.86 (1.75–1.98)
Mental diagnoses	173 (0.22)	1.74 (1.5–2.02)	1.72 (1.48–2.00)	1.48 (1.27–1.72)	1.21 (1.01–1.45)
Substance - rel. disorders^2^	10 (0.5)	3.90 (2.1–7.25)	2.76 (1.48–5.13)	1.98 (1.06–3.71)	1.58 (0.75–3.36)
Affective disorders (F30–F39)	51 (0.19)	1.48 (1.12–1.95)	1.47 (1.11–1.93)	1.17 (0.89–1.55)	0.94 (0.68–1.31)
Neurotic/somatoform disorders*	12 (0.14)	1.12 (0.64–1.97)	1.48 (0.84–2.61)	1.25 (0.71–2.21)	1.05 (0.55–2.03)
Stress - related disorders (F43)	90 (0.25)	1.91 (1.55–2.36)	1.81 (1.47–2.23)	1.67 (1.35–2.05)	1.34 (1.05–1.71)
Other psychiatric disorders	10 (0.28)	2.19 (1.18–4.07)	2.26 (1.22–4.20)	2.00 (1.07–3.72)	1.97 (1.02–3.79)
***Smoking - rel. cancer***					
No sickness absence	2872 (0.06)	1	1	1	1
Non - mental diagnoses^1^	1227 (0.36)	6.79 (6.34–7.27)	5.10 (4.76–5.46)	3.05 (2.83–3.28)	1.59 (1.43–1.77)
Mental diagnoses	63 (0.08)	1.56 (1.21–2.00)	1.63 (1.27–2.09)	1.37 (1.06–1.76)	1.17 (0.87–1.56)
Substance - rel. disorders^2^	7 (0.35)	6.7 (3.19–14.07)	4.2 (2.00–8.82)	2.58 (1.21–5.5)	2.15 (0.87–5.3)
Affective disord. (F30–F39)	15 (0.06)	1.07 (0.64–1.77)	1.11 (0.67–1.84)	0.87 (0.52–1.44)	0.67 (0.36–1.25)
Neurotic/somatoform disorders*	7 (0.08)	1.60 (0.76–3.37)	2.26 (1.08–4.76)	1.83 (0.87–3.84)	1.79 (0.8–4.00)
Stress - rel. disorders (F43)	33 (0.09)	1.72 (1.22–2.43)	1.77 (1.25–2.49)	1.63 (1.16–2.30)	1.45 (0.99–2.12)
Other psychiatric disorders	1 (0.03)	0.54 (0.08–3.81)	0.58 (0.08–4.12)	0.5 (0.07–3.56)	–
***Not Smoking-rel. cancer***					
No sickness absence	4198 (0.09)	1	1	1	1
Non - mental diagnoses^1^	2248 (0.66)	8.55 (8.11–9.01)	6.71 (6.37–7.08)	3.23 (3.06–3.42)	2.01 (1.86–2.17)
Mental diagnoses	110 (0.14)	1.87 (1.54–2.26)	1.79 (1.48–2.16)	1.55 (1.28–1.88)	1.24 (0.99–1.56)
Substance - rel. disorders^2^	3 (0.15)	1.97 (0.64–6.12)	1.51 (0.49–4.68)	1.22 (0.39–3.81)	0.91 (0.22–3.68)
Affective disorders (F30–F39)	36 (0.13)	1.76 (1.27–2.44)	1.71 (1.23–2.37)	1.38 (0.99–1.92)	1.13 (0.76–1.66)
Neurotic/somatoform disorders*	5 (0.06)	0.79 (0.33–1.89)	1.00 (0.42–2.41)	0.87 (0.36–2.11)	0.58 (0.19–1.80)
Stress - related disorders (F43)	57 (0.16)	2.04 (1.57–2.66)	1.85 (1.42–2.41)	1.69 (1.30–2.20)	1.28 (0.94–1.75)
Other psychiatric disorders	9 (0.25)	3.32 (1.73–6.39)	3.34 (1.74–6.43)	3.00 (1.56–5.79)	3.26 (1.69–6.29)

Model 0: Crude; Model 1: Adjusted for age, sex, educational level, family situation, region, country of birth; Model 2: As model 1 and adjusted for inpatient and outpatient care due to mental and cancer diagnoses as well as non-mental/non - cancer diagnoses. Model 3: like model 2 but with a 2 years -wash-out period;

* F40–F42, F44–F49,

1 Including sick-leave spells for which diagnoses are missing;

2 including F10–F19.

### Suicide

Adjusted for sex and age, the risk for suicide was nine-fold increased in case of a new sick-leave spell due to mental diagnoses (Table 3). This risk estimate remained three-fold increased in the multivariate analysis. Sickness absence due to any of the mental diagnoses was associated with very high risks for suicide, reaching HRs of 11.4 and 20.5 for sickness absence due to affective diagnoses and substance-related diagnoses adjusted for age and sex, respectively. Adjustment for additional socio-demographic factors and particularly for health care had strong effects on risk estimates associated with sickness absence due to all diagnoses with the exception of stress-related diagnoses.

### Cancer - smoking and non-smoking Related

In the multivariate analyses, sickness absence due to mental diagnoses was associated with an increase in risk for cancer mortality, generally and smoking and non-smoking related (Table 4). After multivariate adjustment, sickness absence due to substance-related and stress-related diagnoses, respectively, remained associated with an increased risk of cancer mortality and smoking-related cancer mortality. Sickness absence due to stress-related diagnoses and other mental diagnoses was predictive of non-smoking related cancer mortality (Table 4). Employing a two-year wash-out period, sickness absence due to mental diagnoses and specifically due to stress-related diagnoses remained to be associated with an increased risk of cancer mortality (Table 4).

**Table 5:** Crude and adjusted Hazard Ratios (HR) with 95% confidence intervals (CI) for mortality due to circulatory disease in relation to mental and non-mental sick-leave diagnoses, stratified by sex.

Sickness absence in 2005	Death	Model 0	Model 1	Model 2	Model 3	Model 4
	N, %	HR (95% CI)	HR (95% CI)	HR (95% CI)	HR (95% CI)	HR (95% CI)
***Women***						
No sickness absence	1050 (0.05)	1	1	1	1	1
Non-mentaldiagnoses^1^	175 (0.09)	2.03 (1.73–2.38)	1.70 (1.45–2.00)	1.62 (1.38–1.90)	1.43 (1.21–1.69)	1.30 (1.10–1.53)
Mental diagnoses	31 (0.06)	1.39 (0.97–1.98)	1.36 (0.95–1.94)	1.36 (0.95–1.94)	1.31 (0.91–1.87)	1.08 (0.75–1.55)
Substance - related disorders^2^	3 (0.56)	13.16 (4.24–40.87)	11.25 (3.62–34.95)	8.78 (2.82–27.29)	7.71 (2.48–24.01)	2.96 (0.93–9.44)
Affective disorders (F30–F39)	12 (0.07)	1.61 (0.91–2.84)	1.63 (0.92–2.88)	1.57 (0.89–2.77)	1.49 (0.84–2.64)	1.15 (0.64–2.05)
Neurotic/somatoform disorders*	5 (0.09)	2.16 (0.90–5.19)	3.03 (1.26–7.29)	2.79 (1.16–6.73)	2.66 (1.10–6.40)	2.05 (0.85–4.96)
Stress - related disorders (F43)	10 (0.04)	0.88 (0.47–1.64)	0.79 (0.42–1.47)	0.83 (0.44–1.54)	0.81 (0.43–1.50)	0.76 (0.40–1.41)
Other mental disorders	1 (0.04)	1.02 (0.14–7.23)	1.07 (0.15–7.62)	1.02 (0.14–7.23)	0.98 (0.14–6.97)	0.74 (0.10–5.30)
***Men***						
No sickness absence	4074 (0.17)	1	1	1	1	1
Non - mental diagnoses^1^	560 (0.4)	2.71 (2.48–2.96)	1.94 (1.77–2.12)	1.80 (1.65–1.97)	1.48 (1.35–1.62)	1.27 (1.16–1.4)
Mental diagnoses	95 (0.38)	2.62 (2.14–3.21)	2.47 (2.02–3.03)	2.33 (1.9–2.85)	2.11 (1.72–2.59)	1.57 (1.28–1.94)
Substance - related disorders^2^	20 (1.35)	9.44 (6.09–14.66)	7.56 (4.87–11.73)	5.24 (3.37–8.13)	4.15 (2.67–6.45)	1.99 (1.27–3.14)
Affective disorders (F30–F39)	37 (0.39)	2.72 (1.96–3.75)	2.54 (1.84–3.51)	2.37 (1.71–3.27)	2.15 (1.55–2.97)	1.57 (1.13–2.19)
Neurotic/somatoform disorders*	4 (0.14)	0.95 (0.36–2.53)	1.15 (0.43–3.06)	1.02 (0.38–2.73)	0.92 (0.34–2.45)	0.70 (0.26–1.88)
Stress - related disorders (F43)	30 (0.3)	2.07 (1.45–2.97)	1.89 (1.32–2.7)	1.95 (1.36–2.79)	1.85 (1.29–2.65)	1.69 (1.18–2.43)
Other mental disorders	4 (0.32)	2.2 (0.82–5.86)	2.22 (0.83–5.93)	1.93 (0.72–5.15)	1.73 (0.65–4.61)	1.20 (0.45–3.21)

Model 0: Crude; Model 1: Adjusted for age; Model 2: As model 1 and adjusted for educational level, family situation, region, country of birth; Model 3: As model 2 and adjusted for outpatient and inpatient care due to non - mental or non - circulatory diagnoses; Model 4: As model 3 and adjusted for outpatient and inpatient care due to mental diagnoses and due to circulatory diagnoses;

* F40–F42, F44–F49,

1 Including sick-leave spells for which diagnoses are missing;

2 including F10–F19.

### Mortality Due to Circulatory Disease

In the multivariate models, sickness absence due to mental diagnoses was associated with a significant increase (60%) in risk of mortality due to circulatory disease in men, but not in women (Table 5). Among men, sickness absence due to substance-related, stress-related, or affective diagnoses was associated with an increased risk of mortality due to circulatory disease. There were significant gender differences in the association of sickness absence due to mental diagnoses in general and specifically due to stress-related diagnoses with mortality due to circulatory disease in the univariate models (p = 0.001). Only the interaction with gender with regard to the analyses of sickness absence due to stress-related diagnoses and mortality due to circulatory disease remained significant in the multivariate model (p = 0.017).

## Discussion

Sick-leave due to a mental diagnosis was associated with a 70% increase in risk of all-cause mortality for both women and men, after adjustment for socio-demographic variables and inpatient and specialised outpatient health care. This increased risk was also observed for suicide and cancer mortality (all cancers, smoking, and non-smoking related cancer) for both women and men and for death due to circulatory disease in men but not women. Associations with all-cause mortality were found for all mental sickness absence diagnostic groups studied.

The main strengths of this study include the very large and population-based cohort, the prospective design, no loss to follow up, and administrative register data of high quality, [21], [22] which recorded exposure, confounders, and outcome independently from each other. The study included the whole population of working ages in Sweden, which is unique in this research field to date and offered satisfactory statistical power for the analyses of sickness absence due to specific mental diagnoses and rare outcomes like suicide. Moreover, to our best knowledge, this is the first study to include information on diagnosis-specific in- and outpatient hospital care in analyses of sickness absence with mental diagnoses and cause-specific mortality. Still, it should be mentioned that there is some loss of information regarding mental outpatient care [23]. Some limitations of this study should be noted. First, little is known about the validity of the sick-leave diagnoses. The only study carried out in Sweden shows acceptable validity [24]. Due to the remaining stigma of mental diagnoses, [25], [26] we assume a high validity of mental diagnoses on sickness certificates. That shorter sick-leave spells were not included can be seen as both a strength and a limitation. The major part of the shorter spells is not certified by a physician, which means a lower validity. Also, we might have missed some mental sickness absences, as we only had access to the first diagnoses of a sick-leave spell. The relatively short follow-up time may have had an impact on the estimate precision in some analyses particularly those focussing on sickness absence due to specific mental diagnoses in relation to cause-specific mortality.

Incident sickness absence due to mental diagnoses adjusted for a number of socio-demographic factors and previous health care was associated with a 70% increased risk for all-cause mortality, for both women and men. This is comparable to a 90% increased mortality risk in an occupational study, adjusted for age, sex, and employment grade with a longer follow up time [8]. Similarly to earlier studies from other treatment settings, the following mental diagnoses showed the strongest associations with all-cause mortality: developmental and organic disorders, substance-related disorders, and schizophrenia and non-affective psychoses in men [14], [16], [18]. However, it is important to remember that patients included in such studies, differ considerably from ours. We found a significantly higher risk of mortality with sickness absence due to mental diagnoses in general, and particularly with affective disorders in men but not in women. The significant gender difference disappeared after adjustment for previous health-care contacts due to mental diagnoses. Results derived from different treatment settings suggest increased mortality for men compared to women with mental diagnoses, [27] even if contradicting results have been found [28]. We found a 3.4 fold increased risk for suicide in individuals sickness absent due to mental diagnoses after controlling for socio-demographic factors and health care. This is comparable to a previous occupational study, reporting a five–fold increased risk of suicide if sickness absent due to mental diagnoses, adjusted for socio-demographic factors, tobacco, and alcohol use [7]. While a number of studies have analysed the risk of suicide in patients treated for mental disorders in different treatment settings, [17], [18] this is, to our best knowledge, the first study analysing patients sickness absent with specific mental diagnoses and the risk for suicide. Actually, all sick-leave diagnostic groups were strongly associated with an increased risk for suicide. The fact that stress-related diagnoses were the most frequent mental sick-leave diagnoses and the finding that the high risk of suicide in patients sickness absent due to these diagnoses was only marginally affected by controlling for specialised psychiatric hospital care, is of clinical importance for the sickness certifying physician. These results indicate an increased risk of suicide in patients managed outside specialised psychiatric services and that better treatment and follow-up is required for this group of patients to prevent suicide.

We found a 50% increased risk for cancer mortality in relation to sickness absence due to mental diagnoses in the multivariate analyses. These results are in line with one previous occupational cohort study, reporting a two-fold increased risk of cancer mortality in case of mental sickness absence [8]. The increased risk remained elevated even when excluding cancer deaths occurring during the first two years. This finding suggests that the association of mental sickness absence with cancer mortality is not purely an indication of misinterpreting early signs of cancer as mental health complaints [29]. We also found a 40% and 60% increased risk of smoking-related and non-smoking related cancer, respectively. In the above mentioned cohort, [7] the association of sickness absence due to mental disorders with smoking-related cancer failed to remain significant, after controlling for marital status, tobacco smoking, and alcohol use. The association of sickness absence due to mental diagnoses and cancer mortality was mainly driven by substance-related and stress-related diagnoses for cancer mortality and for smoking-related cancer mortality, while sickness absence with affective disorders was related to non-smoking related cancer. These findings are in line with previously reported increased risk of cancer mortality in patients from different treatment settings with alcohol and drug abuse [16], [18] and depression [18], [30]. The association of sickness absence due to stress-related mental diagnoses and cancer mortality has, to our best knowledge, not been reported to date. While it might be difficult to distinguish sickness absence due to these diagnoses from sickness absence due to depression, [31] the association of sickness absence due to stress-related diagnoses and subsequent cancer mortality deserves more detailed scrutiny in future research.

We found an increased risk of death due to circulatory disease only in men sickness absent due to mental diagnoses, particularly due to substance-related, stress-related, or affective diagnoses. A recent study reported an increased risk for death due to circulatory disease if sickness absent due to mental diagnoses, but did not stratify for gender [7]. The literature, based on studies from different treatment settings, is somewhat inconsistent with regard to possible gender differences in the association of mental diagnoses, particularly affective disorders, and mortality due to circulatory disease [14], [16], [17], [32]. Further research is required in order to scrutinise potential gender differences in the association of diagnosis-specific mental sickness absence with mortality due to circulatory disease.

This is the first nationwide cohort study of sickness absence due to specific mental diagnoses and risk of death. This study shows that several specific mental sick-leave diagnoses predicted all-cause and cause-specific mortality, independent from a range of socio-demographic factors and previous and ongoing psychiatric in- and outpatient health care.

Possible mechanisms underlying the association of mental disorders and the risk of cause-specific mortality may range from disparities in access and utilisation of health care, delays in detection, inadequate treatment and follow-up, poor treatment compliance and neglect of physical problem in patients with mental disorders of the treating physician [33], [34], [35], [36], [37], [38], [39]. In addition, health-related behavior often related to mental disorders, like alcohol consumption, smoking, poor diet, and decreased physical exercise, as well as medication side effects may contribute to the excess mortality risk in patients with mental disorders [33], [34], [35], [36], [37], [38], [39].

Knowledge about the prognosis of patients sickness absent with specific mental diagnoses is of crucial clinical importance. As the predominant proportion of patients with mental sick-leave diagnoses are managed in primary health care and the increased risk for cause-specific mortality remained after control for specialised in- and outpatient health care, these findings have considerable implications for health care provision at primary health care level. Sickness absence due to specific mental diagnoses may here be used as a risk indictor for subsequent mortality and tailor-made intervention put in place in time. Interventions focussing on individuals sickness absent due to mental disorders have been proven effective in improving health outcomes in different settings, and could be set in place more broadly [40], [41].
